# Medication Knowledge and Adherence in Type 2 Diabetes Mellitus Patients in Brunei Darussalam: A Pioneer Study in Brunei Darussalam

**DOI:** 10.3390/ijerph19127470

**Published:** 2022-06-18

**Authors:** Najwa Hazwani Muhammad Haskani, Hui Poh Goh, Daniel Vui Teck Wee, Andi Hermansyah, Khang Wen Goh, Long Chiau Ming

**Affiliations:** 1 PAPRSB Institute of Health Sciences, Universiti Brunei Darussalam, Gadong BE1410, Brunei; 18b3082@ubd.edu.bn (N.H.M.H.); long.ming@ubd.edu.bn (L.C.M.); 2 Pharmacy Department, Suri Seri Begawan Hospital, Kuala Belait KA1131, Brunei; daniel.wee@moh.gov.bn; 3 Department of Pharmacy Practice, Faculty of Pharmacy, Universitas Airlangga, Surabaya 60115, Indonesia; 4 Faculty of Data Sciences and Information Technology, INTI International University, Nilai 71800, Malaysia; khangwen.goh@newinti.edu.my

**Keywords:** medication knowledge, medication adherence, type 2 diabetes mellitus, glycemic control, HbA1c

## Abstract

Aim: The present study measured the medication knowledge and medication adherence in patients with type 2 diabetes in Brunei Darussalam. Demographic details and diabetes knowledge were also evaluated. Methods: A cross-sectional study conducted via the administration of a structured questionnaire consisting of 4 sections via a face-to-face interview. Results: A total of 118 participants were interviewed. A majority of the participants were aged 40 years or above (106, 89.8%). The mean number of total medications that the participants were taking was 7.36 ± 2.87 and the mean number of antidiabetic medications was 2.39 ± 1.06. As for the antidiabetic therapy, the largest proportion of the participants were taking oral antidiabetic medications only (87, 73.73%). In the diabetes knowledge section of the questionnaire, more than half of the participants (63, 53.34%) scored higher than the acquired mean score. Family history, education level, and total medications taken were significantly correlated with diabetes knowledge. However, in the medication knowledge section of the questionnaire, the mean score (3.37 ± 1.38) was below the intended score for good knowledge. Medication knowledge has been significantly associated with gender, family history and total medications taken. A majority of the participants reported non-adherence (74, 62.71%) due to various reasons. In this study, those of the Malay race were significantly correlated with adherence to their medication regimen. This study also revealed that there is no significant relationship between diabetes knowledge, medication knowledge and medication adherence. Conclusions: The present study provides insights in regard to patients with type 2 diabetes in Brunei Darussalam and their knowledge towards the disease as well as their medications. Despite the lack of significance between the variables, the rate of non-adherence is still alarming. Further studies are required to better understand the barriers to non-adherence in these patients.

## 1. Introduction

Diabetes mellitus (DM) can be described as a group of metabolic diseases indicated by chronic hyperglycemia resulting from impaired insulin secretion, action or both. There are a number of pathogenic pathways by which diabetes can develop, resulting in different types of DM with the common ones being type 1 diabetes mellitus (T1DM) and type 2 diabetes mellitus (T2DM) [1,2]. T2DM is the most common type of DM; 90% of DM cases are T2DM. It is also known as non-insulin dependent diabetes. By the year 2030, it is estimated that 439 million people will acquire T2DM [3].

According to a study done in 2016 on 2082 participants, the prevalence of diabetes in Brunei Darussalam was 9.7% in which diabetes was indicated as having fasting blood glucose ≥7.0 mmol/L. It has been stated that despite the success in controlling communicable diseases, there was an epidemic of non-communicable diseases (NCDs) in the sultanate, including T2DM. The prevalence of diabetes in Brunei Darussalam is lower in comparison to the neighbouring countries, Singapore and Malaysia, with 11% and 18% of diabetes prevalence, respectively. However, considering that obesity has been linked to be a risk factor for T2DM, the prevalence of obesity in Brunei Darussalam, 28%, ranks highest among Southeast Asian countries. A weighted measurement by the study had confirmed the extensive presence of key risk factors, such as smoking and obesity, and morbidities in relation to NCD in Brunei Darussalam. The standardized methodology used in the study is important for the surveillance of public health interventions as it provides policy makers with the ability to better evaluate public health needs, identify priority settings and set proper and relevant objectives [4]. 

Diabetes mellitus has been the third leading cause of death in Brunei Darussalam since 2012, accounting for 10.1% of the total deaths nationwide in 2017. This is an increase from the 2016 mortality rate from diabetes mellitus, which was 9.1%. Due to the proliferation of diabetes in the sultanate, the government, in particular the Ministry of Health (MOH), has been implementing initiatives to minimize the prevalence of the disease. These initiatives encompass an array of strategies such as the provision of health promotion and health education activities in an attempt to raise diabetes awareness. A health programme administered by the government, known as the Health Screening Effectiveness (3PK) Programme, has been offered to the public. It provides health screening services for risk profiling and early detection of NCDs, including diabetes. Diabetes screening can also be performed in any private and public health centre in the sultanate. Despite the efforts made by the government, there is still the need to involve other relevant stakeholders to cooperate and engage in these pursuits to raise diabetes awareness, empower the people to make healthy decisions, and ultimately control the prevalence of diabetes in Brunei Darussalam [4,5].

Patients’ knowledge of their medications can be defined as the set of necessary information obtained by the patient on their medication for correct use. This includes the therapeutic goal, dosage, times to take it, safety, and its conservation methods in addition to possible interactions and adverse reactions [6]. Poor patient medication knowledge may result in a decrease in medication’s effectiveness, the emergence of other health issues, medication misuse, as well as negative results linked to medication such as adverse drug reaction [7]. Adverse drug reaction is considered as an important public health concern as its incidence is prevalent.

Sound medication knowledge has been demonstrated to be positively corresponded with a better quality of life, treatment adherence, and achieving desirable results in pharmacotherapy. This is therefore crucial in disease management and in combating the frequency of adverse drug reactions [8]. One of the prerequisites for a patient’s involvement in reducing medication error is proper patient education. Despite this, there is not much research that assesses patient medication knowledge in hospitals [9]. A large number of outpatients are subjected to polypharmacy and this can increase the risk of other health issues such as drug interactions and the possibility of toxicity [10]. To further examine the aforementioned issues, the objectives of this project were to evaluate the medication knowledge and medication adherence amongst T2DM patients. This study also aimed to evaluate the correlation among medication adherence and medication knowledge. 

## 2. Methods

### 2.1. Study Design and Settings

The project followed a cross-sectional study design which was conducted from 26 January 2022 until 31 March 2022. The data collection was done via a researcher administered questionnaire in the form of a face-to-face interview (Refer to Supplementary Material). Participants who satisfy the inclusion criteria were approached and upon receiving their consent, the participants were interviewed. The data collection took place at one referral public hospital located in Kuala Belait. 

A total of 135 participants were interviewed for this project with the first 15 participants being the pilot studies. Therefore, a total of 120 participants’ data were analysed for this project. However, data from two participants were discarded due to incomplete details. The data of 118 participants were therefore included in this study. 

### 2.2. Study Population

The minimum required sample size was determined by the use of Raosoft software [11]. The values included in the software include; (1) the population size, which is calculated to be 42,874 (9.7% prevalence of diabetes in Brunei) (2) the error of margin, which is 5% and (3) the confidence level of 95%. According to the values inserted, the required sample size is 381 patients. However, there were factors which resulted in the lower amount of the data collected compared to the required sample size. The hindering factors included; (1) COVID-19 restrictions, (2) caregivers/relatives/friends taking the medicines on behalf of the patients, (3) only conducting the study in one district rather than throughout the entire nation and (4) some patients did not want to be interviewed.

The patients who were approached for this study were those who fulfill the inclusion criteria. The patients were those attending their appointments at the Diabetic Clinic and those who were collecting their medicines at the Outpatient Pharmacy. The patients were identified through their drug profile on the Brunei Darussalam Health Information and Management System (Bru-HIMs). Bru-HIMs is a Brunei Darussalam Ministry of Health’s information technology initiative where the management of all patients’ data, such as patients’ medical records, in public hospitals and health centres are stored electronically by an electronic patient record system [12]. The inclusion criteria were patients aged 18 years or above; residents of Brunei Darussalam (local or permanent resident); and those who have been diagnosed with T2DM for at least 6 months. The exclusion criteria were patients with Type 1 diabetes mellitus; patients not able to understand English and Malay languages; and patients taking antidiabetic medication for non-DM indication. 

### 2.3. Questionnaire and Score Measurement 

The questionnaire was received in English and was then translated to Malay by a language expert and the questionnaire was then translated back for accuracy and clarity. The questionnaire consists of four sections. The first section incorporates questions which analyse the sociodemographic information of patients and their clinical status. The second section assesses the patients’ knowledge on diabetes using the Diabetes Knowledge Questionnaire (DKQ) developed by Garcia et al. [13]. The tool comprises 24 items with 3 answer options; “yes”, “no”, and “I don’t know”. A correct option is awarded with 1 point and no point is awarded for the incorrect option (“I don’t know” is considered as incorrect). The overall score of each patient was calculated by averaging the total points awarded, with the minimum and maximum scores being 0 and 24 respectively. A higher score indicates better diabetes knowledge [13]. 

The third section measures the patient’s knowledge on their antidiabetic medications using the questionnaire adapted by McPherson et al. and Okuyan et al., which was used in the study by Mekonnen, G., and Gelayee, D. [14,15,16]. The section includes seven validated yes/no questions. Total medication knowledge score is determined by the number of correct responses in which 1 point is granted for each correct answer and 0 if incorrectly answered or not answered. An additional one point was granted to each participant if the exact mechanism of their medication was stated correctly for question 2 in the section. Therefore, the maximum score for this section is 8 and the minimum is 0. A good knowledge score is indicated by a score of ≥5 [16]. 

The fourth section evaluates a patient’s medication adherence using the tool devised by experts for a study by Arifulla M et al. [17]. This section consists of questions in regard to adherence and related factors. Medication adherence was reported via a yes or no question. 

### 2.4. Pilot Study

First, face and content validation were conducted among six senior researchers who had conducted a similar study earlier. Second, a pilot study was conducted with the first 15 randomly selected participants who fulfilled the inclusion criteria. The pilot study data were analysed using Cronbach’s Alpha test on RStudio software [18,19]. For the diabetes knowledge questionnaire consisting of 24 items, the Cronbach’s Alpha was calculated to be α = 0.74. The medication knowledge section consisted of 7 items with the Cronbach’s Alpha of α = 0.70. Originally, the medication adherence questionnaire consisted of 11 questions. However, based on the results of the pilot study, the questionnaire was revised by removing 3 items: “Do you make your own modification in the dose of medicines prescribed?”, “Do you make your own modification in the timing of the prescribed medications?”, and “Do you have good knowledge about antidiabetic medications prescribed to you?”. The medication adherence final questionnaire consisted of 8 items and the Cronbach’s Alpha is α = 0.61. Third, the test-retest reliability was also performed for which we have reported a good consistency of the questionnaires with a Cronbach’s Alpha value of >0.5 [20]. Fourth, the revised questionnaire was then again re-validated by the six senior researchers to ensure its contents were accurate and consistent. 

### 2.5. Data Analysis

The data collected were analysed using RStudio software. Demographic and clinical variables were described using descriptive statistics. Frequency counts and percentages were used to present the responses to categorical variables. A Pearson correlation test was used to test the association between the demographic variables and medication knowledge as well as between medication knowledge and medication adherence. The significance level was set at *p* < 0.05. 

### 2.6. Ethical Approval

Ethical approvals for the project were obtained from PAPRSB Institute of Health Sciences Research Ethics Committee, Universiti Brunei Darussalam. Patients who participated in the project were briefed on the project and its aim. They were also required to fill in a consent form prior to the interview and those who refused to do so were not eligible to participate in this survey. The data collected from this project had remained confidential throughout the period of this project. As for the questionnaires, permissions had been obtained from the authors of corresponding questionnaires. 

## 3. Results

### 3.1. Demographic Data

The study included data from a total of 118 participants in which their demographic details are tabulated in Table 1. The mean number of total medications that the participants were taking at the time is 7.36 ± 2.87 (95% CI: 6.83, 7.88). The mean number of antidiabetic medications that the participants were taking is 2.39 ± 1.06 (95% CI: 2.20, 2.58). Most of the participants were taking only oral antidiabetic medications for their antidiabetic regimen (87, 73.73%).

### 3.2. Diabetes Knowledge Questionnaire

The participants’ scores for each question are tabulated in Table 2. It can be seen that questions 1, 3, 4, 7, 9 and 17 were answered incorrectly by most participants. Based on question 1, almost all participants (113, 95.76%) had a misconception that excessive consumption of sugar and sweet foods can result in diabetes. A large number of participants (79, 66.95%) thought that diabetes was caused by failure of the kidneys to keep sugar out of the urine. Almost as many participants (77, 62.25%) believed that diabetes was curable if they maintained a healthy lifestyle paired with a healthy diet and adhering to their medications. Many participants (71, 60.17%) thought that doing a urine test was the best way to check their diabetes. Fewer participants (47, 39.83%) were confused that sweating and shaking are signs of hypoglycaemia, and 41 (34.75%) were uncertain or did not know that frequent urination and thirsts are signs of hyperglycaemia.

**Figure 1:**
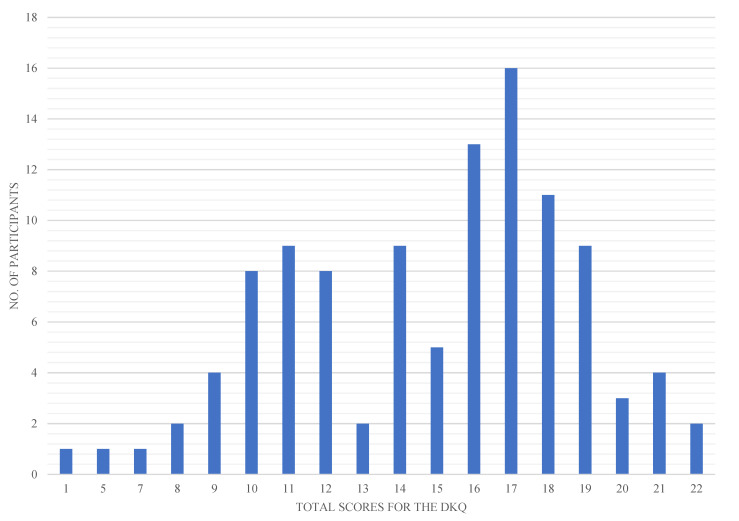
Distribution of number of participants and their total scores for the diabetes knowledge questionnaire.

The mean score for the DKQ is 14.69 (SD= 3.81). The maximum score for DKQ is 24 where 0 is the minimum. A higher score in the DKQ indicates better knowledge of the disease. As seen in Figure 1, the distribution is skewed to the right which demonstrates that more participants scored higher than the mean score, suggesting that there are more participants with above average diabetes knowledge.

### 3.3. Medication Knowledge

A majority of the participants were unable to name their antidiabetic medications (102, 46.44%). Slightly fewer of the participants (50, 42.37%) did not know the indication of their antidiabetic medications. Most participants were able to demonstrate the correct way to take their antidiabetic medications (87, 73.73%), which include correct dose, correct frequency and correct route of administration. Almost all participants knew the correct timing for their antidiabetic medications (113, 95.76%). However, it was discovered that 82 (69.49%) participants did not know of any possible side effects of their antidiabetic medications. Nevertheless, more than half of the participants knew what to do in case they experience any side effects (73, 61.86%). The question “Do you know what to do if you miss a dose of your medication(s)?” was not included in the calculation for the score of medication knowledge, however it was discovered that 94 (79.66%) knew what to do in the case of missing a dose of their antidiabetic medications. 

**Figure 2:**
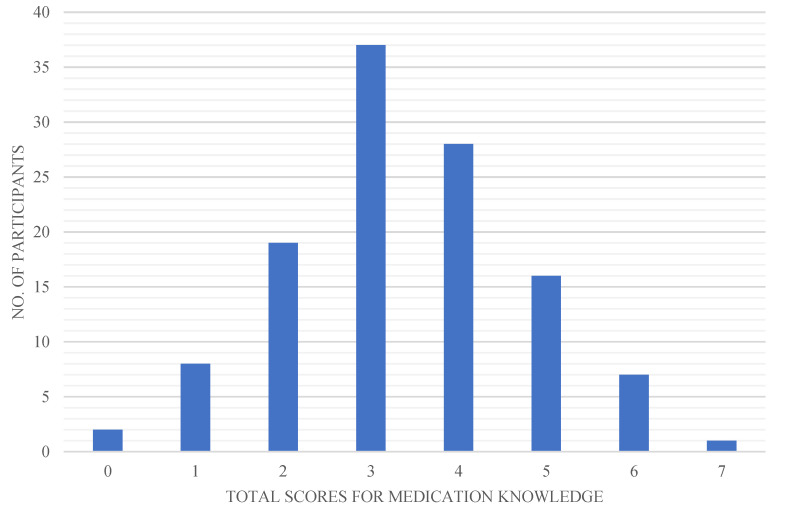
Distribution of the number of participants and their scores for the medication knowledge questionnaire.

The maximum score for the medication knowledge questionnaire was 7 and the minimum was 0. The mean score for this questionnaire was 3.37 (SD = 1.38) and the distribution for medication knowledge is shown in Figure 2. 

### 3.4. Medication Adherence

A large proportion of the participants admitted that they did not adhere to their antidiabetic therapy due to various reasons (74, 62.71%). A majority of the non-adherent participants revealed that forgetfulness was the culprit (64, 86.49%). A few of them stated that side effects were the cause of their non-adherence (12, 16.22%). Some also had reasons other than the one listed in the questionnaire (16, 21.62%) which included: no stock of medications at home, not taking medications and/or reducing the dose when feeling better, busy schedule, bitter taste, and not wanting to rely on medications. 

Most participants did not monitor their blood glucose regularly (89, 75.42%). Some claimed that this was due to the cost burden of the needles which were single use only. Due to COVID-19 restrictions, borders to other countries were closed which hindered the participants from purchasing the needles from those countries with a lower currency rate. Almost all participants claimed to know the importance of their antidiabetic medications (116, 98.31%). Only a small proportion of the participants claimed that their doctor did not provide information on diabetes (23, 19.49%), but the information was given to them by their dietitian and/or nurses. However, more than half revealed that their doctor did not give information on their antidiabetic medications (56, 47.46%). This is because the doctor told them that the pharmacists and/or dispensers would counsel them on their medications so the doctor did not give any further details regarding their medications. Most participants were not involved in their treatment decision (71, 60.17%). For those who were involved in the treatment decisions, it was mainly in regard to the initiation of insulin therapy. Participants were asked whether they were willing to be started on insulin and this is where their decision is involved. Almost all participants reported that they felt comfortable asking questions to their doctors in regard to their health conditions and/or medications (105, 88.98%). 

One of the aims of this study was to evaluate the relationship between demographic variables, diabetes knowledge, medication knowledge and medication adherence. Their correlation has been measured as tabulated in Table 3. 

## 4. Discussion

The study was carried out to measure the medication knowledge and medication adherence among patients with T2DM in Brunei Darussalam regarding their antidiabetic medications. Diabetes mellitus is the third leading cause of death in Brunei Darussalam with a prevalence of 9.7% [4] In 2017, the disease contributed to 10.1% of the total deaths in the sultanate. Due to the extensive nature of diabetes within Brunei Darussalam, one of the initiatives taken to minimize patients’ exposure to unwanted complications of diabetes is to ensure their adherence to antidiabetic medication [21]. 

For the diabetes knowledge questionnaire, a majority of the participants thought that excessive sugar intake can result in diabetes (113, 95.76%). Albeit the fact that consuming sweets can raise the blood glucose level, diabetes is a metabolic disorder in which severe hyperglycaemia is one of its markers [1]. The misconception for this question may be due to its vagueness. Lifestyle changes have been associated with increased risk of developing type 2 diabetes. Due to this, participants might have misconstrued the concept of excessive consumption of sweets leading to diabetes [22]. Participants also demonstrated that they thought the kidneys played an important role in the development of diabetes. This misconception can be due to the fact that kidney disease is a common yet dangerous complications in people with type 2 diabetes [23]. A number of participants agreed that testing the urine is the best way to check for diabetes (71, 60.17%). This could be due to the fact that some participants claimed that they can estimate the state of their glucose level by observing their urine upon urinating in which foamy urine indicates that their glucose level is on the higher end [24]. There were several participants who were confused with signs of hypoglycaemia and hyperglycaemia (39.83% and 34.75% respectively). Knowing the signs of hypoglycaemia and hyperglycaemia is important in patients with diabetes as these would guide them into making the right and safe decision, i.e to take their medications or to consume more sweets [25]. 

A cross-sectional study was conducted in three health centres in Pakistan utilizing the Starr County Diabetes Knowledge Questionnaire in the Urdu language. The study revealed that diabetes knowledge was significantly correlated with the participants’ gender, level of education, family history of diabetes and their antidiabetic therapy (*p* < 0.05) [26]. Based on a self-administered questionnaire-based study done in Malaysia by Abbasi et al. in 2018, it was discovered that the largest proportion of the participants (47.7%) exhibited moderate levels of diabetes knowledge. The diabetes knowledge was tested using the Translated Michigan Diabetes Knowledge Test (MDKT). In this study, factors that were significantly associated with diabetes knowledge included age, race, education level, occupation and the nature of the antidiabetic therapy [27,28]. As seen in Table 3, diabetes knowledge has been shown to be significantly correlated with education level, family and total medications taken. The higher the education level that the participants received, the higher they scored on the diabetes knowledge questionnaire (*p* = 0.048, *r* = 0.183). This trend can also be supported by the studies done by Bukhsh et al. (2019) and Abbasi et al. (2018) [26,27]. Family history of diabetes has shown a significant inverse correlation with diabetes knowledge history (*p* = 0.019, *r* = −0.215). This result can be interpreted as participants with known family history of diabetes to have better knowledge of the disease which was also observed by Bukhsh et al. (2019) [26]. There is also a significant relationship between the number of medications that the participants were taking and their diabetes knowledge score (*p* = 0.045, *r* = 0.185). Participants who were taking more medications scored higher on the diabetes knowledge questionnaire. 

As for medication knowledge, a good level of knowledge is indicated by a score of ≥5. The mean score for the medication knowledge was 3.37 ± 1.38, which suggests that most of the participants have a subpar level of knowledge in regard to their antidiabetic medications. Only 24 participants (20.34%) participants had a score of ≥5. Most participants (102, 86.44%) were not able to name all of their antidiabetic medications and 82 participants (69.49%) were not aware of any possible side effects that may be caused by their antidiabetic medications. As polypharmacy is common amongst the participants with the mean of total medications taken of 7.36 ± 2.87, it is not surprising that most of the participants were not able to name all of their antidiabetic medications as well as their side effects. However, as hypoglycemia is a familiar phenomenon to those taking antidiabetic medications, it is important for patients to acknowledge this orthodox side effect in order for them to correct it appropriately. Effective communication between the doctor and patient is important to tackle the risk of hypoglycemia due to possible misinterpretation of the therapy and to reduce episodes of hypoglycemia [29]. A small group of the participants did not know the correct way to take their antidiabetic medications. It was observed that the majority of those who answered wrongly were still following their old medication regimen prior to the changes. The wrong administration may result in medication misuse and adverse drug reactions [30]. Gender, family history of diabetes and total medications taken have a significant effect on the participants’ medication knowledge score. Women have shown to have better knowledge of their medications in comparison to men (*p* = 0.022, *r* = 0.211). Participants with family history of diabetes scored higher on the medication knowledge questionnaire as opposed to those with no known background (*p* = 0.019, *r* = −0.215). Having a higher number of total medications taken have resulted in participants scoring lower on the medication knowledge questionnaire (*p* = 0.001, *r* = 0.315). 

In this study, non-adherence was observed in a majority of the participants (74, 62.71) with forgetfulness being the main culprit (64, 86.49%). As a lot of the patients were on polypharmacy, receiving excessive information on their medications may result in difficulties to retain vital information, thus leading to forgetfulness [31]. Medication adherence is only significantly correlated with race (*p* = 0.011, *r* = −0.233). The negative correlation here implies that those who are of non-Malay race are more likely to be non-adherent to their regimen. This could be due to language barrier or cultural differences. A study conducted in Iran which studied medication adherence amongst patients with type 2 diabetes using the Morisky Medication Adherence Scale (MMAS-8) had observed moderate adherence for most of the participants (59.12%) whereas 27.2% of the participants showcased low adherence to their medication regimen. Age was one of the significant factors for medication adherence in the study [21]. 

This study revealed that there is no significant association between diabetes knowledge, medication knowledge and medication adherence. A study in 2018 also demonstrated the same hypothesis whereby no significant correlation had been discovered between diabetes knowledge and medication adherence [32]. However, there have been studies conducted which support the notion that diabetes knowledge and medication adherence are significantly correlated. As such, one of the studies in 2020 discovered that there is a positive correlation, albeit weak, between diabetes knowledge and medication adherence in patients with type 2 diabetes (*p* < 0.01) [33]. A study in 2011 also revealed that subpar knowledge on diabetes was associated with a lower rate of medication adherence [34]. Furthermore, two studies found that medication knowledge is a significant predictor for medication adherence (*p* < 0.001) [15,16]. In this present study, there is a lack of significant correlation between medication knowledge and medication adherence. Various factors may have resulted in this outcome. Some participants with below satisfactory medication knowledge had shown adherence to their medications as their medication regimen had been planned out through a pill box or through the help of family members. Furthermore, due to the absence of a financial burden on the participants’ side in obtaining their medications, this may have played a role in the participants’ medication adherence. 

The reported mean rate of non-adherence in developed countries is only 50% whereas the rate is higher in developing countries. In this study, the rate of non-adherence was 62.71% which concurs with the hypothesis as Brunei Darussalam is a developing country [35]. Nevertheless, initiatives should be taken in order to ameliorate adherence in these patients. One of the incentives was setting a reminder for patients to take their medications, especially as forgetfulness was the main reason for non-adherence in this present study. The reminder method can be done through various ways such as via text messages and mobile phone applications [36,37]. Another intervention which has been proven to promote adherence is personalized patient education, such as informing patients regarding their individualized risks presuming non-adherence to their treatment [36]. Multifaceted and personalized pharmacist interventions, such as simplifying treatment regimes, have been shown to be effective in aiding patients to adhere to their medications [38]. Strengths, limitations and implications for practice.

The whole sample was interviewed by the same researcher which ensured that the consistency of data collection was maintained. Furthermore, since the interview was administered with the assistance of a single researcher, this allowed participants to clarify any questions that needed clarity. 

This study, like other studies, also has some inherent limitations. This study relies on the self-reporting method for the medication adherence questionnaire which may result in recall bias and lack of transparency, thus affecting the actual rate of medication adherence. Moreover, the study was done in only one hospital in Brunei Darussalam which may have affected the scope of the participants. However, this study is able to generalize to the whole Brunei because the included sample was representative of the demographic of the Bruneian population. The present study also lacks access to more sociodemographic de-tails such as BMI, socioeconomic status, nutritional status, and glucose level. The participants in this study were selected through a convenience sampling which may have resulted in selection bias. 

## 5. Conclusions

Medication knowledge and medication adherence amongst T2DM patients in Brunei Darussalam were examined in this study. It was discovered that a majority of the patients had subpar knowledge regarding their antidiabetic medications. Additionally, it was revealed that almost two-thirds of patients were non-adherent to their T2DM medication. It was discovered that diabetes knowledge was significantly associated with education level, family history and total medications taken. Medication knowledge was significantly linked with gender, family history and total medications taken. Medication adherence was significantly correlated with race. There was, however, no significant correlation between diabetes knowledge, medication knowledge and medication adherence. Despite the lack of correlation, the level of medication knowledge and rate of non-adherence were considerably high. There have been strategies implemented with the aim to mitigate these issues, including the use of mobile phone applications for reminders and personalized patient education. Nonetheless, review and augmentation of these strategies are required in order to maximize their efficacy and efficiency. For future studies, assessing further relevant components, for instance BMI, blood glucose level and diet, may provide greater insights and understanding in regard to the correlation between medication knowledge and adherence.

## Figures and Tables

**Table 1:** Sociodemographic details of participants (*n* = 118).

Details	*n* (%)
**Age (year)**	
<40	12 (10.20)
40–60	61 (51.70)
>60	45 (38.10)
**Gender**	
Male	62 (52.50)
Female	56 (47.50)
**Race**	
Malay	101 (85.60)
Non-malay	17 (14.40)
**Education level**	
No formal education	2 (1.70)
Primary school	13 (11.00)
Secondary school	80 (67.80)
Higher education	23 (19.50)
**Occupation**	
Retired	45 (38.14)
Unemployed	23 (19.49)
Private sector	23 (19.49)
Government sector	21 (17.80)
Self-employed	5 (4.24)
Student	1 (0.85)
**Family history**	
Yes	88 (74.58)
No	24 (20.34)
Not sure	6 (5.08)
**Duration since diagnosed with T2DM**	
6–11 months	2 (1.69)
1–4 years	25 (21.19)
5–9 years	16 (13.56)
≥10	75 (63.56)
**Participants’ antidiabetic therapy**	
Insulin only	2 (1.69)
Insulin combined with oral medication	29 (24.58)
Oral medication only	87 (73.73)
Abbreviation:	
T2DM–Type 2 diabetes mellitus	

**Table 2:** Number of participants with correct answers to the questions in diabetes knowledge questionnaire.

Questions	*n* (%)
1. Eating too much sugar and other sweet foods is a cause of diabetes.	5 (4.24)
2. The usual cause of diabetes is lack of effective insulin in the body.	77 (65.25)
3. Diabetes is caused by failure of the kidneys to keep sugar out of the urine.	39 (33.05)
4. Kidneys produce insulin.	33 (28.21)
5. In untreated diabetes, the amount of sugar in the blood usually increases.	114 (96.61)
6. If I am diabetic, my children have a higher chance of being diabetic.	83 (70.34)
7. Diabetes can be cured.	41 (34.75)
8. A fasting blood sugar level of 11.7 mmol/L is too high.	109 (92.37)
9. The best way to check my diabetes is by testing my urine.	47 (39.83)
10. Regular exercise will increase the need for insulin or other diabetic medication.	80 (67.80)
11. There are two main types of diabetes: Type 1 (insulin-dependent) and Type 2 (non-insulin-dependent).	71 (60.17)
12. An insulin reaction (severe hypoglycemia) is caused by too much food.	97 (82.20)
13. Medication is more important than diet and exercise to control my diabetes.	58 (49.15)
14. Diabetes often causes poor circulation.	69 (58.47)
15. Cuts and abrasions on diabetics heal more slowly.	98 (83.05)
16. Diabetics should take extra care when cutting their toenails.	105 (88.98)
17. A person with diabetes should cleanse a cut with iodine and alcohol.	29 (24.58)
18. The way I prepare my food is as important as the foods I eat.	92 (77.97)
19. Diabetes can damage my kidneys.	109 (92.37)
20. Diabetes can cause loss of feelings in my hands, fingers, and feet.	90 (76.27)
21. Shaking and sweating are signs of high blood sugar.	71 (60.17)
22. Frequent urination and thirst are signs of low blood sugar.	77 (65.25)
23. Tight elastic hose or socks are not bad for diabetics.	65 (55.08)
24. A diabetic diet consists mostly of special foods.	75 (63.56)

**Table 3:** The significant relationship between the variables.

	*p*-Value and CI	Correlation Coefficient
**Diabetes knowledge with:**		
Education level	<0.05 (95% CI: 0.002, 0.352)	0.183
Family history	<0.05 (95% CI: −0.381, −0.036)	−0.215
Total medications taken	<0.05 (95% CI: 0.004, 0.354)	0.185
**Medication knowledge with:**		
Gender	<0.05 (95% CI: 0.031, 0.377)	0.211
Family history	<0.05 (95% CI: −0.227, 0.133)	−0.215
Total medications taken	<0.05 (95% CI: −0.469, −0.143)	−0.315
**Medication adherence with:**		
Race	<0.05 (95% CI: −0.397, −0.05)	−0.233

## Supplementary Materials

The following supporting information can be downloaded at: https://www.mdpi.com/article/10.3390/ijerph19127470/s1. Supplementary Information: Data collection form [13]. Table S1. Diabetes Knowledge Questionnaire [16,17,13]. Table S2. Diabetes Medication Knowledge Questionnaire. Table S3. Diabetes Medication Adherence Questionnaire.
