# Supplementary material for: Medication Knowledge and Adherence in Type 2 Diabetes Mellitus Patients in Brunei Darussalam: A Pioneer Study in Brunei Darussalam
Source: Int J Environ Res Public Health. 2022 Jun 18;19(12):7470. doi: 10.3390/ijerph19127470 (PMC9224316; doi:10.3390/ijerph19127470)
Supplement: Supplementary file 1 [file ijerph-19-07470-s001.zip › ijerph-1742959-supplementary.pdf]

Supplementary Information: Data collection form
Reference no.:

GENERAL INFORMATION

MAKLUMAT AM

Age/Umur

❑ <40 years old/tahun

❑ 40–60 years old/tahun

❑ >60 years old/*tahun*

1. **Gender**/*Jantina*

❑ Male/*Lelaki*
❑ Female/*Perempuan*

2. **Race**/*Bangsa*

❑ Malay/*Melayu*
❑ Chinese/*Cina*
❑ Indian/*India*
❑ Others (*lain-lain):* please specify/*sila nyatakan*

3. **Educational Level/***Peringkat Pendidikan*

❑ No formal education/*Tiada pendidikan formal*
❑ Primary school/*Sekolah rendah*
❑ Secondary school/*Sekolah menengah*
❑ Higher education/*Pendidikan tinggi*

4. Occupation/*Pekerjaan*

❑ Unemployed/*Tidak bekerja*
❑ Student/*Pelajar*
❑ Government sector/*Sektor kerajaan*
❑ Private sector/*Sektor swasta*
❑ Self-employed/*Bekerja sendiri*
❑ Retired/*Bersara*

5. **Do you have a family history of diabetes?/***Adakah anda mempunyai sejarah keluarga yang menghidap kencing manis?*

❑ Yes/*Ya* ❑ No/*Tidak*
❑ Not sure/*Kurang pasti*

6. **How long have you been diagnosed with diabetes?/***Berapa lama semenjak anda didiagnos dengan kencing manis?*

❑ Less than a year/*tahun*
❑ 1–4 years/*tahun*
❑ 5–9 years/*tahun*
❑ ≥10 years/*tahun*

7. **How many medications are you currently taking?/***Berapa jumlah ubat yang anda ambil sekarang?*

medicines/*ubat*

8. **How many antidiabetic therapy are you taking?/***Berapa jumlah ubat kencing manis yang anda ambil sekarang?*

medicines/*ubat*

9. What is your antidiabetic therapy?/*Apakah rawatan kencing manis anda?*

❑ Insulin only/*Insulin sahaja*
❑ Insulin combined with oral medication/*Insulin dan ubat oral kencing manis*
❑ Oral medication only/*ubat oral kencing manis sahaja*

KNOWLEDGE ON DIABETES
PENGETAHUAN MENGENAI DIABETES

[1]

Table S1. Diabetes Knowledge Questionnaire.

**Questions**<br>***Soalan***	**Yes**<br>***Ya***	**No**<br>***Tidak***	**I Don't Know**<br>***Kurang pasti***
1. Eating too much sugar and other sweet foods is a cause of diabetes.<br>*Memakan gula dan makanan manis secara berlebihan boleh menyebabkan kencing manis.*			
2. The usual cause of diabetes is lack of effective insulin in the body.<br>*Kencing manis adalah biasanya disebabkan oleh kekurangan insulin yang berkesan didalam badan.*			
3. Diabetes is caused by failure of the kidneys to keep sugar out of the urine.<br>*Kencing manis adalah disebabkan oleh kegagalan buah pinggang daripada mencegah gula masuk ke dalam air kencing.*			
4. Kidneys produce insulin.<br>*Buah pinggang menghasilkan insulin.*			
5. In untreated diabetes, the amount of sugar in the blood usually increases.<br>*Jika kencing manis tidak dirawat, jumlah gula dalam darah biasanya meningkat.*			
6. If I am diabetic, my children have a higher chance of being diabetic.<br>*Sekiranya saya menghidap kencing manis, anak saya mempunyai peluang lebih tinggi untuk menghidap kencing manis.*			
	**Yes**<br>*Ya*	**No**<br>*Tidak*	**I don't know**<br>*Kurang pasti*
7. Diabetes can be cured.<br>*Kencing manis dapat disembuhkan.*			
8. A fasting blood sugar level of 11.7 mmol/L is too high.<br>*Tahap gula darah berpuasa 11.7 mmol/L adalah terlalu tinggi.*			
9. The best way to check my diabetes is by testing my urine.<br>*Cara terbaik untuk memeriksa kencing manis saya adalah dengan menguji air kencing saya.*			
10. Regular exercise will increase the need for insulin or other diabetic medication.<br>*Senaman secara kerap akan meningkatkan keperluan insulin atau ubat kencing manis.*			

11. There are two main types of diabetes: Type 1 (insulin-dependant) and Type 2 (non-insulin-dependant).
Terdapat dua jenis diabetes yang utama: Jenis 1 (bergantung kepada insulin) dan Jenis 2 (tidak bergantung kepada insulin).

12. An insulin reaction (severe hypoglycemia) is caused by too much food.
Terlalu banyak makanan boleh menyebabkan reaksi insulin (paras gula dalam darah yang terlampau rendah).

13. Medication is more important than diet and exercise to control my diabetes.
Ubat adalah lebih penting daripada diet dan senaman untuk mengawal kencing manis saya.

14. Diabetes often causes poor circulation.
Kencing manis sering menyebabkan peredaran darah yang lemah.

	Yes<br>*Ya*	No<br>*Tidak*	I don't know<br>*Kurang pasti*
15. Cuts and abrasions on diabetics heal more slowly.<br>*Luka dan melecet pada pesakit kencing manis sembuh dengan lebih perlahan.*			
16. Diabetics should take extra care when cutting their toenails.<br>*Pesakit kencing manis perlu berhati-hati apabila memotong kuku kaki.*			
17. A person with diabetes should cleanse a cut with iodine and alcohol.<br>*Pesakit kencing manis harus membersihkan luka dengan iodin dan alkohol.*			
18. The way I prepare my food is as important as the foods I eat.<br>*Cara saya menyediakan makanan (goreng, rebus) adalah sama pentingnya dengan jenis makanan yang saya makan.*			
19. Diabetes can damage my kidneys.<br>*Kencing manis boleh merosakkan buah pinggang saya.*			
20. Diabetes can cause loss of feelings in my hands, fingers, and feet.<br>*Kencing manis boleh menyebabkan kehilangan sensasi di tangan, jari dan kaki saya.*			
21. Shaking and sweating are signs of high blood sugar.<br>*Menggigil dan berpeluh adalah tanda paras gula dalam darah yang tinggi.*			

	**Yes**<br>***Ya***	**No**<br>***Tidak***	**I Don't Know**<br>***Kurang pasti***
22. Frequent urination and thirst are signs of low blood sugar.<br>*Kerap buang air kecil dan kerap dahaga adalah tanda paras gula dalam darah yang rendah.*			
23. Tight elastic hose or socks are not bad for diabetics.<br>*Stokin anjal yang ketat tidak mempunyai kesan buruk kepada pesakit diabetes.*			
24. A diabetic diet consists mostly of special foods.<br>*Diet pesakit kencing manis kebanyakannya terdiri daripada makanan yang istimewa. [1]*			

KNOWLEDGE ON ANTIDIABETIC MEDICATIONS

PENGETAHUAN MENGENAI UBAT-UBATAN KENCING MANIS

[2]

Table S2. Diabetes Medication Knowledge Questionnaire

**Questions**<br>***Soalan***	**Yes**<br>***Ya***	**No**<br>***Tidak***
Can you list the name of antidiabetic medication(s) you are currently taking?<br>*Bolehkah anda menyenaraikan nama ubat kencing manis yang anda sedang ambil?*		
Can you tell me why you are taking the antidiabetic medication(s)?<br>*Bolehkah anda beritahu mengapa anda mengambil ubat kenicng manis?*		
Do you know how to take the medication(s)?<br>*Adakah anda tahu cara mengambil ubat-ubatan anda?*		
Do you know when to take the medication(s)?<br>*Adakah anda tahu bila anda perlu makan/ambil ubat-ubatan anda?*		
Do you know the possible side effects of the medication(s)?<br>*Adakah anda tahu kemungkinan kesan sampingan ubat-ubatan anda?*		
Do you know what to do if your medication's side effects occur?<br>*Adakah anda tahu apa yang harus dilakukan sekiranya kesan sampingan ubat-ubatan anda berlaku?*		
Do you know what to do if you miss a dose of your medication(s)?<br>*Adakah anda tahu apa yang perlu dilakukan jika anda terlepas satu dos ubat anda?*		

ANTIDIABETIC MEDICATION ADHERENCE
PEMATUHAN TERHADAP UBAT-UBATAN KENCING MANIS

[3]
Do you take the prescribed antidiabetic medications as advised by your doctor?
Adakah anda mengambil ubat-ubatan kencing manis seperti yang diarahkan oleh doktor anda?
❑ Yes/*Ya* ❑ No/*Tidak*
If No, please tick the options below (/)
Jika Tidak, sila tandakan sebab yang berkaitan dengan anda (/)

Table S3. Diabetes Medication Adherence Questionnaire.

	**Yes**	**No**		**Yes**	**No**
Lack of finance/*Masalah kewangan*			Side effects/*Kesan sampingan*		
Feeling medication is not effective/*Saya rasa ubat tidak berkesan*			Feeling the dose given is high/*Terasa dos yang diberikan adalah tinggi*		
Interferes with my meal plan/*Mengganggu pelan permakanan saya*			Complexity of medication regimen/*Rejimen ubat yang rumit*		
Taking them since many tears/*Mengambil ubat-ubatan sejak bertahun-tahun*			Multiple medications/*Mengambil ubat-ubatan yang banyak*		
I forget/*Saya lupa*			Poor family support/*Kurang sokongan keluarga*		
Others/*lain-lain:*					

Items	Yes	No
Do you regularly monitor your blood glucose?<br>*Adakah anda sentiasa memantau paras gula dalam darah anda?*		
Do you know the importance of your medications?<br>*Adakah anda tahu kepentingan ubat-ubatan anda?*		
Did your doctor give information on diabetes?<br>*Adakah doktor anda memberi maklumat tentang penyakit kencing manis?*		
Did your doctor give information antidiabetic medications?<br>*Adakah doktor anda memberi maklumat tentang ubat kencing manis anda?*		
Were you involved in treatment decisions?<br>*Adakah anda terlibat dalam membuat keputusan untuk rawatan anda?*		
Do you feel comfortable asking questions to your doctors?<br>*Adakah anda berasa selesa bertanya soalan kepada doktor anda?*		

Reference

[1] Garcia, A., Villagomez, E., Brown, S., Kouzekanani, K., and Hanis, C. (2001). The Starr County Diabetes Education Study: Development of the Spanish-language diabetes knowledge questionnaire. *Diabetes Care*, *24*(1), 16-21. doi: 10.2337/diacare.24.1.16

[2] Mekonnen, G., and Gelayee, D. (2020). Low Medication Knowledge and Adherence to Oral Chronic Medications among Patients Attending Community Pharmacies: A Cross-Sectional Study in a Low-Income Country. *Biomed Research International*, *2020*, 1-8. doi: 10.1155/2020/4392058

[3] Arifulla M, John L, Sreedharan J, Muttappallymyalil J, Basha S. Patients' Adherence to Anti-Diabetic Medications in a Hospital at Ajman, UAE. Malays J Med Sci. 2014;21(1):44-49.
